# Supplementary material for: Concomitant medication, comorbidity and survival in patients with breast cancer
Source: Nat Commun. 2024 Apr 5;15:2966. doi: 10.1038/s41467-024-47002-3 (PMC10997660; doi:10.1038/s41467-024-47002-3)
Supplement: Supplementary file 1 — Supplementary Information [file 41467_2024_47002_MOESM1_ESM.pdf]

Concomitant medication, comorbidity and survival in patients with breast cancer

Supplementary Information

Supplementary Methods **3**
Data source and study population *3*
Concomitant medication *3*
Comorbid conditions *5*
Other covariates *6*
Pre-exposure covariates 6
Post-exposure covariates 9
Causal inference pipeline and methods used *9*
Step 1: Adjustment by inverse probability of treatment weighting (IPTW) 10
Step 2: Adjustment quality check 11
Step 3: Average treatment effect estimation 12
Step 4: Mediation analyses 12
Step 5: Kaplan-Meier survival curves 15
Software *15*
Supplementary Tables **17**
Supplementary Table 1: Results of sensitivity analyses. 17
Supplementary Table 2: List of ATC classes discarded from the analyses. 19
Supplementary Table 3: Medication presentation according to the medication packaging form. 20
Supplementary Figures **22**
Supplementary Figure 1: Frequency and number of comorbid conditions for the total population, by deprivation index and by age at BC diagnosis. 22
Supplementary Figure 2: Association between comorbid conditions (at disease level). 24
Supplementary Figure 3: Screenshot of the web page for visualizing the associations between comorbid conditions within the ADRENALINE application (https://adrenaline.curie.fr/static/network_comor/index.html). 25

Supplementary Figure 4: Association between medications (at ATC level 2) and comorbid conditions (at category level). 26

Supplementary Figure 5: Frequency and number of medications used at the time of BC diagnosis, for the total population, by deprivation index and by age at BC diagnosis. 28

Supplementary Figure 6: Association between medications (at ATC level 2). 30

Supplementary Figure 7: Screenshot of the web page for visualizing associations between medications at ATC level 5 within the ADRENALINE web application (https://adrenaline.curie.fr/static/network/index.html). 31

Supplementary Figure 8: Association between the number of comorbid conditions and the number of medications. 32

Supplementary Figure 9: Simplified flowchart of the French Early Breast Cancer Cohort. 33

Supplementary Figure 10: Modified ATC codes and ATC names for ATC class G03 (sex hormones and modulators of the genital system). 34

Supplementary Figure 11: Directed acyclic graph (DAG). 35

Supplementary Figure 12: Directed acyclic graph (DAG) for the mediation analyses. 37

Supplementary References 39

Supplementary Methods

Data source and study population

Demographic data, hospital discharge reports and outpatient care data were available for the year preceding patient inclusion, up to December 31, 2018. Data for the patients' history of long-term illness (LTI) were available until December 31, 2018.

The diagnosis codes were recorded in the SNDS, based on the International Classification of Diseases – 10th revision, ICD-10[1]. Procedures were recorded with the CCAM classification (Classification Communes des Actes Médicaux). Medications prescribed in outpatient care were recorded with CIP (Code Identifiant de Présentation) codes. In hospital, only costly innovative drugs included in a special reimbursement process called "list en sus" were recorded, in the form of UCD (Unités Communes de Dispensation) codes. Both the UCD and CIP codes were linked to the ATC classification (Anatomical Therapeutic and Chemical classification) of the World Health Organization. Medical devices reimbursed by the French health insurance system (external prosthetics, orthotics, active implantable medical devices, invalid carriages, medical beds etc.) were recorded with LPP (Liste des produits et prestations) codes. In outpatient care, the type of medical service (teleconsultation, nursing care, dental care, etc.) was recorded with NGAP (Nomemenclature Générale des Actes Professionnels) codes and the physician's specialty was recorded with an untitled nomenclature (variable PSE_SPE_COD).

Concomitant medication

The definition used to identify three months of full treatment was set at the scale of individual medications and depended on presentation and dose schedule.

- *Presentation* was binned into two categories: (1) identifiable individual units (*e.g.* box of pills, single-use syringe); (2) unidentifiable individual units (*e.g.* syrup bottle, tube of cream). Medication packaging was determined from the information (pill, solution, etc.) available in the SNDS database (Supplementary Table 3). Medications for which packing data were missing, or for which the packaging form was insufficient to determine the presentation category were manually checked by a pharmacologist.
- *Dose schedule* was defined as the recommended number of days between two units. If the medication was supposed to be administered several times per day, the dose schedule was set to 1. If the medication was supposed to be administered less frequently than once yearly, the dose schedule was set to 365. If several dose schedules existed for a particular medication, we selected the dose schedule corresponding to the largest number of days. If dose schedule depended on the patient's characteristics, such as weight or age, it was set as unknown. Dose schedules were determined manually by a pharmacologist.

At the patient scale, we defined three months of full treatment with a medication during the six months preceding BC diagnosis as one of the following:

- At least X identifiable individual units of the medication with a dose schedule of less than 180 days delivered in the six months preceding BC diagnosis, where $X = C\left(\frac{90}{\text{dose schedule}}\right)$ and C is the ceiling function;
- At least one identifiable individual unit of the medication with a dose schedule strictly greater than 180 days in the year preceding BC diagnosis;
- At least two pharmacy deliveries of the medication as unidentifiable individual units or with an unknown dose schedule;

- At least Y identifiable individual units of the medication with dose schedule below 180 days and one pharmacy delivery of the medication as unidentifiable individual units or with an unknown dose schedule in the six months preceding BC diagnosis; where $Y = C\left(\frac{45}{\text{dose schedule}}\right)$ and C is the ceiling function.

Comorbid conditions

Following a literature review, the list of comorbid conditions considered was deduced from the union of all diseases used as covariates in 10 articles[2–11]. Overlapping categories were checked manually by a clinician who indicated the category and label to be used. The final list of comorbid conditions included 52 diseases (Supplementary Data), grouped into 12 categories: (1) cardiovascular, (2) endocrine and metabolism, (3) frailty, (4) gastrointestinal, (5) immune, (6) kidney, (7) liver, (8) neurologic, (9) psychiatric disorders, (10) pulmonary, (11) rheumatologic disease and connective tissue disorders, and (12) other. The medical codes used in the 10 articles to identify the defined diseases in the 10 articles were aggregated into a single table. Several types of medical code were used: (1) ICD-10 diagnosis codes (ICD10), (2) ICD-10 diagnosis codes specific of long-term illness (ICD10 (specific for LTI)), (3) CCAM codes for medical procedures (CCAM), (4) LPP codes for medical devices (LPP), (5) NABM codes for biological acts (NABM), (6) NGAP codes for medical procedures performed by non-medical health practitioners (NGAP). Codes relating to medication deliveries (CIP and UCD) were not retained, to ensure that comorbid conditions were defined independently of exposure to medication. For a given comorbid conditions, the medical codes retained in only one article of the 10 listed above were manually checked by a clinician; whereas medical codes used in at least two articles were automatically validated. The final list of medical codes is given in Supplementary Data. A given comorbid condition was suspected at the time of BC diagnosis if: (1) there was at least one NABM, CCAM or LPP procedure

code associated with the given comorbid condition in the year preceding the date of BC diagnosis, (2) there was at least one LTI related to an ICD10 code associated with the given comorbid condition during the year preceding BC diagnosis, (3) there was at least one hospital discharge report containing an ICD10 diagnosis code associated with the comorbid condition concerned between 365 days before and up to 180 days after the date of BC diagnosis (we used 180 days after diagnosis to include the comorbid conditions noted by the surgeon at the time of first surgery for BC), or (4) the comorbid condition considered was "Frailty (proxy)" and the patient had at least 150 days of home nursing care (NGAP code "AIS") in the year preceding BC diagnosis. Sensitivity analyses were conducted to evaluate the impact of comorbid conditions identification timing on the results. Two additional timeframes for diagnosis codes in hospital discharge reports were tested: (i) the year before BC diagnosis up to BC surgery, and (ii) the one-year period before BC surgery; resulting in two alternative definitions of comorbid conditions. The causal inference pipeline was then re-run with the modified definitions of comorbid conditions for the sixteen molecules that were identified as being associated with either OS, DFS, or both.

Other covariates

Pre-exposure covariates

(1) *Age at BC diagnosis: Age at BC diagnosis* was available directly from the FRESH database. It was calculated as the rounded difference, in years, between the date of BC diagnosis and date of birth[12], and was included as a continuous variable in the propensity score models.

(2) *Deprivation index of the area of residence:* The *area of residence was defined* as the zip code of the French '*département*' (equivalent to a county) of residence at the time of first BC surgery. We used the 'FDep09' geographic socioeconomic index as a measure of social deprivation, as described elsewhere[13]. This index was defined at the

'*commune*' (the smallest administrative unit in France) level in 2009, exclusively for mainland France. The *patient's deprivation index* was set as the mean 'FDep09' index for the '*communes*' included in the patient's *département* of residence in mainland France. It was set as 'missing' for overseas *départements*. The *deprivation index* was classified into six categories: (1) "Overseas *départements*"; and the five quintiles of the distribution for patients living in mainland France: (2) "1st quintile (least deprived)", (3) "2nd quintile", (4) "3rd quintile", (5) "4th quintile" and (6) 5th quintile (most deprived). It was included as a categorical variable in the propensity score models.

(3) Number of general practitioner (GP) visits in the year preceding BC diagnosis: General practitioner (GP) visits were identified by outpatient care visits with specialist code "1" (general practice), "22" (general practice specialist with diploma) or "23" (general practice specialist acknowledged by the French Medical Board). The *number of GP visits in the year preceding BC diagnosis* was calculated as the number of days with at least one GP visit during the 365 days preceding BC diagnosis. This variable was binned into the following categories: (1) 0 (no GP visits in the year preceding BC diagnosis); (2) 1 (one visit in the year preceding BC diagnosis); (3) 2-3 (two or three visits in the year preceding BC diagnosis); (4) 4+ (4 or more visits in the year preceding BC diagnosis).

(4) Number of gynecologist visits in the year preceding BC diagnosis: Gynecologist visits were identified by outpatient care visits with specialist code "7" (obstetric gynecologist), "70" (medical gynecologist), "77" (obstetrician) or "79" (obstetric and medical gynecologist). The *number of gynecologist visits in the year preceding BC diagnosis* was calculated as the number of days with at least one gynecologist visit among the 365 days preceding BC diagnosis. The variable was binned into the

following categories: (1) 0 (no gynecologist visit in the year preceding BC diagnosis); (2) 1 (one visit in the year preceding BC diagnosis); (3) 2-3 (two or three visits in the year preceding BC diagnosis); (4) 4+ (4 or more visits in the year preceding BC diagnosis).

(5) *Performance of a mammographic screening in the year preceding BC diagnosis:* screening mammograms were identified based on the presence of the procedure code QEQK004 in both hospital and outpatient care records in the year preceding breast cancer diagnosis. The variable was binned into the following categories: 'yes' if the patient underwent a screening mammography in the year preceding breast cancer diagnosis, or 'no' otherwise.

(6) *Total number of medications to which the patient was exposed at the time of medication:* this variable was defined as the total number of medications other than the medication considered to which the patient was chronically exposed at diagnosis. The total number of medications to which the patient was exposed at the time of medication was classified into four categories: (1) "0"; (1) "1-2", (3) "3-5", and (4) "6+". It was included as a categorical variable in the propensity score models.

(7) *Exposure to other medications*: this variable was calculated as several binary variables, including: (A) exposure to medications in the second-level ATC classes other than that of the medication of interest, (B) exposure to medications in the other third-level ATC classes corresponding to the second-level ATC class of the medication of interest, (C) exposure to medications in the other fourth-level classes corresponding to the third-level ATC class of the medication of interest and (D) exposure to medications in the other fifth-level ATC classes corresponding to the fourth-level ATC class of the medication of interest. As an illustration, for exposure to other medications for A02AA01 we considered: (A) all ATC second-level classes other than A02; (B) A02B

and A02X; (C) A02AB, A02AC, A02AD, A02AF, A02AG, A02AH and A02AX; and (D) A02AA02, A02AA03, A02AA04, A02AA05, A02AA10. As with the medication of interest, only chronic exposure in the six months prior to BC diagnosis was considered. Exposure to ATC classes to which fewer than 300 patients were exposed was not included in the list of variables.

Post-exposure covariates

Post-exposure covariates were directly available from the database[12]. Briefly, BC subtype was inferred from the BC treatments received, as (A) luminal, (B) TNBC (triple-negative breast cancer), (C) HER2-positive (human epidermal growth factor receptor 2-positive), and (D) undefined (if the patient was treated exclusively by surgery with or without radiotherapy). Nodal status (node-positive/negative), and BC treatments (chemotherapy, endocrine therapy) were classified in a binary manner.

Causal inference pipeline and methods used

We denote as A the exposure (concomitant medication); $X = X_1, \dots, X_n$ the confounding variables (pre-exposure covariates), Y the outcome (overall survival or disease-free survival), M_1 the inferred BC subtype and M_2 the nodal status (mediators). A is a binary variable, taking a value of 0 for unexposed patients and 1 for exposed patients. The potential outcome Y^k is defined as the outcome that would have been observed had the patient been exposed to level k. For each patient, only one potential outcome is observed (the potential outcome for the current exposure level). $X \perp Y \mid Z$ indicates that X is independent of Y conditional on Z. We denote a and a^* as the two levels of exposure to be compared (here $a^* = 1$ and $a = 0$ as exposure is binary). For each subject, the effect of treatment is $Y^{a^*} - Y^a$. The average treatment effect at population level (ATE) is defined as $ATE = E\left(Y^{a^*} - Y^a\right)$.

Step 1: Adjustment by inverse probability of treatment weighting (IPTW)

For each patient, the propensity score (PS) was defined as the probability of being exposed to the given medication conditional on pre-exposure covariates (confounders)[14].

$$PS = Pr(A = 1 | X)$$

The PSs were estimated by a weighted logistic regression in which exposure was regressed on all pre-exposure covariates (sociodemographic covariates, comorbid conditions and other concomitant medications). The model was weighted by the inverse of the exposure distribution (one over the frequency of the given class) to account for the strong imbalance in the exposure distribution (in most cases, the proportion of the patients exposed to the medication was very small).

IPTW involves weighting the whole population to balance the distribution of the confounding variables between the exposed and unexposed patients[15]. The weight for each patient was defined as the inverse of the probability of the exposure received by the patient. We used stabilized weights to prevent the weights from becoming very large when PS values were very close to 0 or 1. These stabilized weights were calculated as follows:

$$w = \frac{A \times Pr\ (A = 1)}{PS} + \frac{(1 - A) \times Pr\ (A = 0)}{1 - PS}$$

IPTW weights were trimmed at 5%, by recoding all weights outside the 2.5th and 97.5th percentiles with the values of the 2.5th and 97.5th percentiles, respectively, as advised elsewhere[16].

Step 2: Adjustment quality check

The estimation of ATE by IPTW is dependent on three causal assumptions: (i) the single unit treatment values assumption (SUTVA), according to which there is only one version of treatment and no interference between patients ($Y^k = Y$ when the observed exposure is k), (ii) conditional ignorability, according to which the set of measured confounders is sufficient to adjust for confounding bias ($Y^k \perp A|X$), and (iii) conditional positivity, according to which all patients have a non-zero probability of being exposed to each level of treatment (0<Pr (A = k)<1). We assumed SUTVA and conditional ignorability for all medications but validated conditional positivity *a posteriori* by checking adjustment quality by calculating the standardized mean difference (SMD) in the weighted dataset for each of the confounding variables. For continuous variables, SMD is derived as follows:

$$SMD = \frac{\mu_{a^*}}{\mu_a}\sqrt{\frac{\sigma^2_{a^*} + \sigma^2_a}{2}}$$

where μ_{a^*} is the mean of the covariate in treated individuals ($A = a^*$), μ_a is the mean of the covariate in controls ($A = a$), $\sigma^2_{a^*}$ is the standard deviation of the covariate in treated individuals, and σ^2_a is the standard deviation of the covariate in controls. For categorical variables, SMD is estimated at each level, as follows:

$$SMD = \frac{p_{a^*} - p_a}{\sqrt{\frac{p_{a^*}(1 - p_{a^*}) + p_a(1 - p_a)}{2}}}$$

where p_{a^*} is the (weighted) proportion of the given level among treated individuals and p_a is the (weighted) proportion of the given level among controls. Adjusted SMDs were calculated by weighting the mean and standard deviation with the inverse probability of treatment weights.

Provided that the conditional positivity assumption is satisfied and the model to estimate the propensity score is properly specified, the weighted SMDs should be zero for all confounding covariates. In accordance with published results[14], the adjustment quality was considered insufficient if any SMD had an absolute value above 0.1, in which case the medication was discarded from subsequent analyses.

Step 3: Average treatment effect estimation

We used Wald tests calculated with robust covariances to draw statistical inferences about the estimated hazard ratio (HR). The threshold for statistical significance was p = 0.05.

Step 4: Mediation analyses

Medications with a significant ATE were selected for mediation analyses, which involved breaking down the ATE into several pathways passing through two potential mediators, BC subtype and nodal status (Supplementary Fig. 11). PSEs were estimated by a weighting approach[17] and are expressed as percentages of the total effect (which may be negative). M_1^a denotes the counterfactual value for BC subtype that would have been observed had the exposure been set to level a; $M_2^{a,\mathrm{b}}$ is the counterfactual value for nodal status that would have been observed had the exposure been set to level a and the BC subtype set to level b; and $Y^{a,\mathrm{b},\mathrm{c}}$ is the counterfactual value for the outcome that would have been observed had the exposure been set to level a, the BC subtype set to level b and the nodal status to level c.

We assumed BC subtype and nodal status to be causally related. Standard direct and indirect effects were not, therefore, directly identifiable for each mediator[18]. It was, nevertheless, possible to break the ATE down into three path-specific effects (PSEs): (1) the effect through pathways involving neither a difference in BC subtype nor in nodal status (direct effects); (2)

the effect through pathways involving a difference in nodal status only (effect through node); (3) the effect through pathways involving a difference in BC subtype (and potentially involving a difference in nodal status; effect through subtype). Path-specific effects (PSEs) were estimated as described by Vanderweele[17]. PSE estimation was based on five assumptions: (1) the counterfactual M_1^a, M_2^{a,M_1^a}, and $Y^{a,M_1^a,M_2^{a,M_1^a}}$ equal M_1, M_2 and Y when the observed exposure is a (assumption of consistency); (2) $Y^{a,b,c} \perp A \mid X$ (no unmeasured outcome-exposure confounder); (3) $Y^{a,b,c} \perp (M^1, M^2) \mid A, X$ (no unmeasured outcome-mediator confounder); (4) (M_1^a, M_2^{a,M_1^a}) $\perp A \mid X$ (no unmeasured mediator-exposure confounder) and (5) $Y^{1,b,c} \perp$ (M_1^0, M_2^{0,M_1^0}) $\mid X$ (cross-world independence assumption, satisfied if there is no unmeasured confounder of the joint mediator (M_1, M_2) and the outcome affected by exposure). We will assume that each of these five assumptions holds.

Under these assumptions, the effect through pathways involving neither BC subtype nor nodal status (direct effect) was defined as:

$$\text{Direct effect} = E\left(Y^{1,M_1^0,M_2^{0,M_1^0}} - Y^{0,M_1^0,M_2^{0,M_1^0}}\right).$$

The effect through pathways involving nodal status only (effect through node) was defined as

$$\text{Effect through node} = E\left(Y^{1,M_1^0,M_2^{1,M_1^0}} - Y^{1,M_1^0,M_2^{0,M_1^0}}\right).$$

The effect through pathways involving BC subtype (and potentially involving nodal status, denoted as effect through subtype) was defined as

$$\text{Effect through subtype} = E\left(Y^{1,M_1^1,M_2^{1,M_1^1}} - Y^{1,M_1^0,M_2^{1,M_1^0}}\right).$$

We observe that:

$$\text{Direct effect } + \text{Effect through node } + \text{Effect through subtype}$$

$$= E\left(Y^{1,M_1^1,M_2^{1,M_1^1}} - Y^{0,M_1^0,M_2^{0,M_1^0}}\right) = E(Y^1 - Y^0) = ATE.$$

Thus, the sum of the direct effect, the effect through nodal status and the effect through subtype equals the total effect.

PSEs were estimated by a weighting method described in detail elsewhere[17]. We first merged three copies of the dataset to which we added the "counterfactual exposure variables" A^* and A^{**}. A^* was set to A for the first two replications and to $1 - A$ for the third replication. A^{**} was set to A for the first replication and to $1 - A$ for the second and third replications. We then computed a weight for each row of this new dataset:

$$w = \frac{Pr(M^1|A^*,X) \times Pr(M^2|M^1,A^{**},X)}{Pr(A|X) \times Pr(M^1|A,X) \times Pr(M^2|M^1,A,X)}.$$

The direct effect is estimated as the HR for A in a weighted Cox model regressing Y on A, fitted for patients with $A^* = A^{**} = 0$. The effect through node is estimated as the HR for A^* of a weighted Cox model regressing Y on A^*, fitted for patients with $A = 1$ and $A^{**} = 0$. The effect through subtype is estimated as the HR for A^{**} of a weighted Cox model regressing Y on A^{**}, fitted for patients with $A = 1$ and $A^* = 1$. Confidence intervals were obtained by bootstrapping (50 repetitions).

In addition to path-specific HR, the percentage of the total effect attributable to each path was calculated as follows:

$$\frac{100 * \log(PSE)}{\log(\text{ATE})},$$

where PSE took the value of the Cox hazard ratio obtained for direct effect, effect through subtype or effect through node. This pseudopercentage could take negative values or values greater than 100 if the total effect could be broken down into PSEs with different signs.

Step 5: Kaplan-Meier survival curves

Weighted Kaplan-Meier survival curves were compared with an adjusted log-rank test[19]. The threshold for statistical significance was set at $p = 0.1$, due to the low power of adjusted log-rank tests[20]. The Benjamini-Hochberg multiple testing procedure was applied to all statistical tests performed, including subgroup analyses for BC subtype, nodal status, chemotherapy status, and endocrine therapy status.

Software

Propensity scores were computed with the *glm* function from the R base package stats. Standardized mean differences (SMDs) were computed with the *bal.tab* function from the R package cobalt (version 4.1.0). Cox proportional hazards models were fitted with the *coxph* function from the R package survival (version 3.2-3). Weighted Kaplan-Meier estimators were obtained with the R package RISCA (version 0.8.2). Survival curves were drawn with the R package survminer (version 0.4.7).

The web application was built with Flask version 2.0.2, using Python version 3.9.1.

Supplementary Tables

Supplementary Table 1: Results of sensitivity analyses. Estimated average treatment effect (ATE, Cox hazard ratio) for overall survival (OS) and disease-free survival (DFS), along with its 95% confidence interval, and p-value for the 16 medications significantly associated with OS, DFS, or both, after adjustment for multiple testing, obtained with two alternative timeframes for the use of diagnosis codes in hospital discharge to identify comorbid conditions. We used two-sided Wald tests with robust covariances for statistical inference. No adjustment for multiple comparisons was made at this stage of the pipeline. *(vaginal or transmucosal)

ATC code	Molecule	Overall survival						Disease-free survival					
		Hospital discharge reports up to BC surgery (sensitivity analysis 1)			Hospital discharge reports pertaining to the 1-year period prior to BC surgery (sensitivity analysis 2)			Hospital discharge reports up to BC surgery (sensitivity analysis 1)			Hospital discharge reports pertaining to the 1-year period prior to BC surgery (sensitivity analysis 2)		
		HR	*95% CI*	*P*	*HR*	*95% CI*	*P*	*HR*	*95% CI*	*P*	*HR*	*95% CI*	*P*
A02BC04	Rabeprazole	0.77	0.65 - 0.91	2.158842e-03	0.77	0.65 - 0.91	2.195847e-03	0.84	0.72 - 0.99	3.405849e-02	0.84	0.72 - 0.99	3.500805e-02
A03AX08	Alverine	0.79	0.67 - 0.92	2.420743e-03	0.79	0.67 - 0.92	2.246826e-03	0.86	0.77 - 0.97	1.158576e-02	0.86	0.77 - 0.97	1.136688e-02
B03AA02	Ferrous fumarate	1.71	1.25 - 2.33	7.210579e-04	1.70	1.25 - 2.32	7.210161e-04	1.38	1.07 - 1.78	1.468258e-02	1.37	1.06 - 1.77	1.547688e-02
C07AB03	Atenolol	0.74	0.62 - 0.88	8.426483e-04	0.74	0.63 - 0.88	8.455132e-04	0.80	0.66 - 0.95	1.293721e-02	0.79	0.66 - 0.95	1.254294e-02
C10AA01	Simvastatin	0.73	0.61 - 0.88	1.028553e-03	0.73	0.6 - 0.88	8.45205e-04	0.76	0.63 - 0.92	5.501827e-03	0.76	0.63 - 0.92	4.968454e-03
C10AA07	Rosuvastatin	0.64	0.55 - 0.75	3.08569e-08	0.64	0.55 - 0.75	2.184199e-08	0.72	0.63 - 0.82	1.07842e-06	0.72	0.63 - 0.82	8.912143e-07
G03JA05	Estriol*	0.55	0.4 - 0.75	1.79207e-04	0.55	0.4 - 0.75	1.732795e-04	0.77	0.62 - 0.96	1.947101e-02	0.77	0.62 - 0.96	1.923632e-02
G03KC01	Nomegestrol	0.40	0.26 - 0.61	1.84932e-05	0.40	0.26 - 0.62	2.501424e-05	0.73	0.59 - 0.9	3.380206e-03	0.73	0.59 - 0.91	4.679704e-03
H02AB06	Prednisolone	1.77	1.18 - 2.66	5.70612e-03	1.77	1.18 - 2.66	5.9349e-03	1.65	1.19 - 2.29	2.426888e-03	1.65	1.19 - 2.29	2.691233e-03
H03BB01	Carbimazole	1.27	0.95 - 1.7	1.04905e-01	1.27	0.95 - 1.7	1.048807e-01	1.45	1.14 - 1.85	2.879329e-03	1.45	1.14 - 1.85	2.78257e-03
J01FG01	Pristinamycin	1.90	1.39 - 2.61	6.013807e-05	1.90	1.38 - 2.6	6.727279e-05	1.65	1.25 - 2.17	3.870899e-04	1.64	1.25 - 2.16	4.242708e-04
N05BA04	Oxazepam	1.30	1.15 - 1.47	2.422258e-05	1.30	1.15 - 1.48	2.367675e-05	1.22	1.09 - 1.36	7.026809e-04	1.22	1.09 - 1.36	6.923283e-04
N05BA12	Alprazolam	1.07	0.98 - 1.18	1.44468e-01	1.07	0.97 - 1.18	1.577624e-01	1.12	1.04 - 1.2	2.30708e-03	1.12	1.04 - 1.2	2.485021e-03
N05BB01	Hydroxyzine	1.21	1.07 - 1.38	3.215996e-03	1.21	1.07 - 1.38	3.274543e-03	1.18	1.06 - 1.31	2.442714e-03	1.18	1.06 - 1.31	2.511346e-03
N06AX03	Mianserin	1.36	1.14 - 1.62	5.790888e-04	1.35	1.14 - 1.61	7.245282e-04	1.23	1.05 - 1.44	1.079401e-02	1.23	1.05 - 1.44	1.194082e-02
S01KA02	Hypromellose	0.77	0.62 - 0.94	1.059597e-02	0.77	0.62 - 0.94	1.0988e-02	0.77	0.65 - 0.91	1.745259e-03	0.77	0.65 - 0.91	1.801362e-03

Abbreviations: ATC: Anatomical Therapeutic Chemical; BC: Breast Cancer; HR: Hazard Ratio; CI: Confidence Interval

Supplementary Table 2: List of ATC classes discarded from the analyses.

ATC code	ATC name	Reason to discard
B05B	I.V. solutions	No systemically active molecule
B05XA03	Sodium chloride	No systemically active molecule
D02AA	Silicone products	No systemically active molecule
D02AC	Soft paraffin and fat products	No systemically active molecule
D02AD	Liquid plasters	No systemically active molecule
D02AE	Carbamide products	No systemically active molecule
D02AF	Salicylic acid preparations	No systemically active molecule
D02AX	Other emollients and protectives	No systemically active molecule
D08	Antiseptics and disinfectants	No systemically active molecule
G01AX11	Povidone-iodine	No systemically active molecule
L01	Antineoplastic agents	Cancer treatment
L02	Endocrine therapy	Cancer treatment
L03AX03	BCG vaccine	Cancer treatment
S01XA20	Artificial tears and other indifferent preparations	No systemically active molecule
V04	Diagnostic agents	Diagnostic agent
V07	All other non-therapeutic products	No systemically active molecule
V08	Contrast media	Diagnostic agent

Abbreviations: ATC: Anatomical Therapeutic Chemical.

Supplementary Table 3: Medication presentation according to the medication packaging form.

Packaging form (English)	Packaging form (French)	Presentation
Dressing	*PANSEMENT*	Identifiable individual units
Film	*FILM*	
Granule	*GRANULE*	
Gum	*GOMME*	
Hard gelatine capsule	*GELULE*	
Implant	*IMPLANT*	
Lyophilizate	*LYOPHILISAT*	
Pastille	*PASTILLE*	
Pill	*PILULE*	
Plaster	*EMPLATRE*	
Rectal suppository	*SUPPOSITOIRE*	
Soft gelatin capsule	*CAPSULE MOLLE*	
Tablet	*COMPRIME*	
Transdermal patch	*DISPOSITIF*	
Vaginal suppository	*OVULE*	
Collyrium	*COLLYRE*	Unidentifiable individual units
Cream	*CREME*	
Dressing gauze	*COMPRESSE*	
Emulsion	*EMULSION*	
Foam	*MOUSSE*	
Gel	*GEL*	
Inhalation	*GAZ*	
Lotion	*LOTION*	
Mouthwash	*BAIN DE BOUCHE*	
Nail polish	*VERNIS*	
Ointment	*POMMADE*	
Paste	*PATE*	
Shampoo	*SHAMPOOING*	
Solvent	*SOLVANT*	
Syrup	*SIROP*	
Granules	*GRANULES*	Manually checked at medication scale
Powder	*POUDRE*	
Solution	*SOLUTION*	
Suspension	*SUSPENSION*	
Missing		

Medications for which the packaging form was not sufficient to decide on a presentation

category were manually checked by a pharmacologist for classification as either “Identifiable individual units” or “Unidentifiable individual units.

Supplementary Figures

Supplementary Figure 1: Frequency and number of comorbid conditions for the total population, by deprivation index and by age at BC diagnosis.

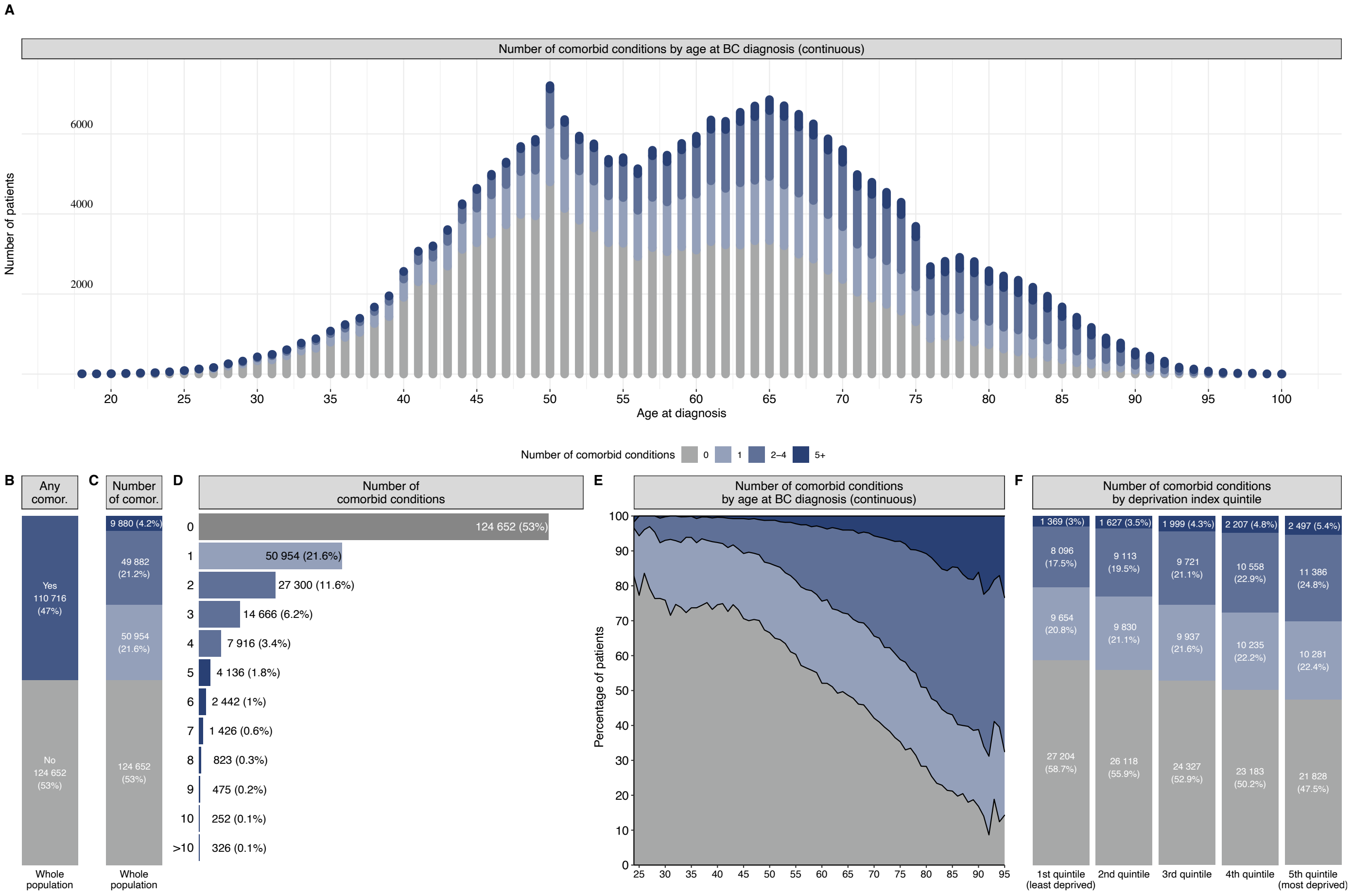

(A) Number of comorbid conditions, by age at BC diagnosis; (B) Presence of a comorbid condition at BC diagnosis in the total population; (C) Number of comorbid conditions, by class, for the total population; (D) Number of comorbid conditions in the total population; (E) Number of comorbid conditions, by age at BC diagnosis (as a percentage); (F) Number of comorbid conditions by deprivation index quintile. Patients from overseas départements for whom deprivation index quintile data were missing (n=4,198) are not displayed on the figure. Source data are provided as a Source Data file. Abbreviations: comor.: comorbid condition(s); BC: breast cancer

Supplementary Figure 2: Association between comorbid conditions (at disease level).

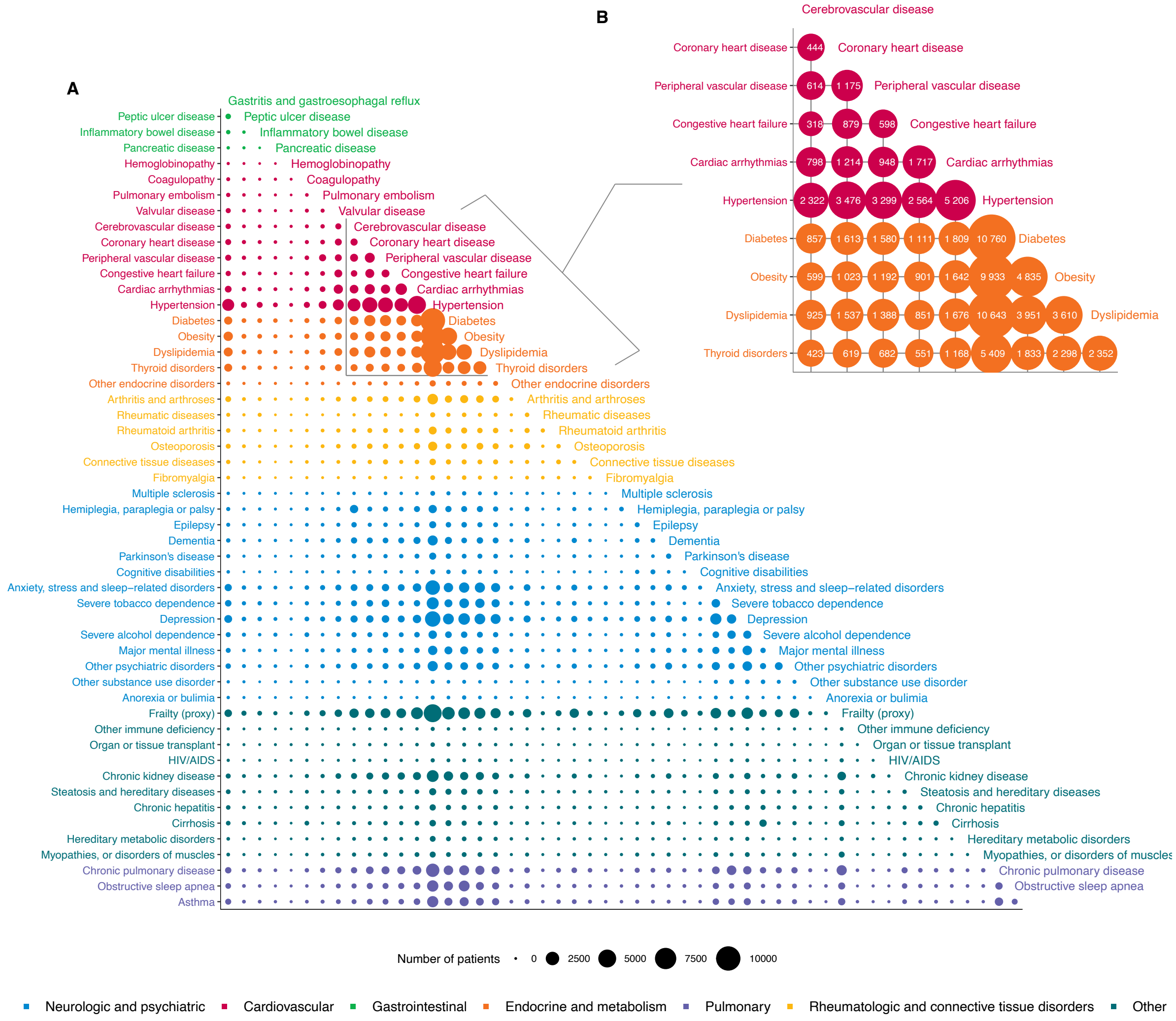

(A) Association between all diseases. The size of the circle is proportional to the number of patients suffering from both comorbid conditions. Diseases are color-coded by category. (B) Associations between several selected frequent cardiovascular and endocrine diseases, with numbers of patients. Source data are provided as a Source Data file. *Abbreviations: HIV: human immunodeficiency virus, AIDS: acquired immunodeficiency syndrome.*

Supplementary Figure 3: Screenshot of the web page for visualizing the associations between comorbid conditions within the ADRENALINE application (https://adrenaline.curie.fr/static/network_comor/index.html).

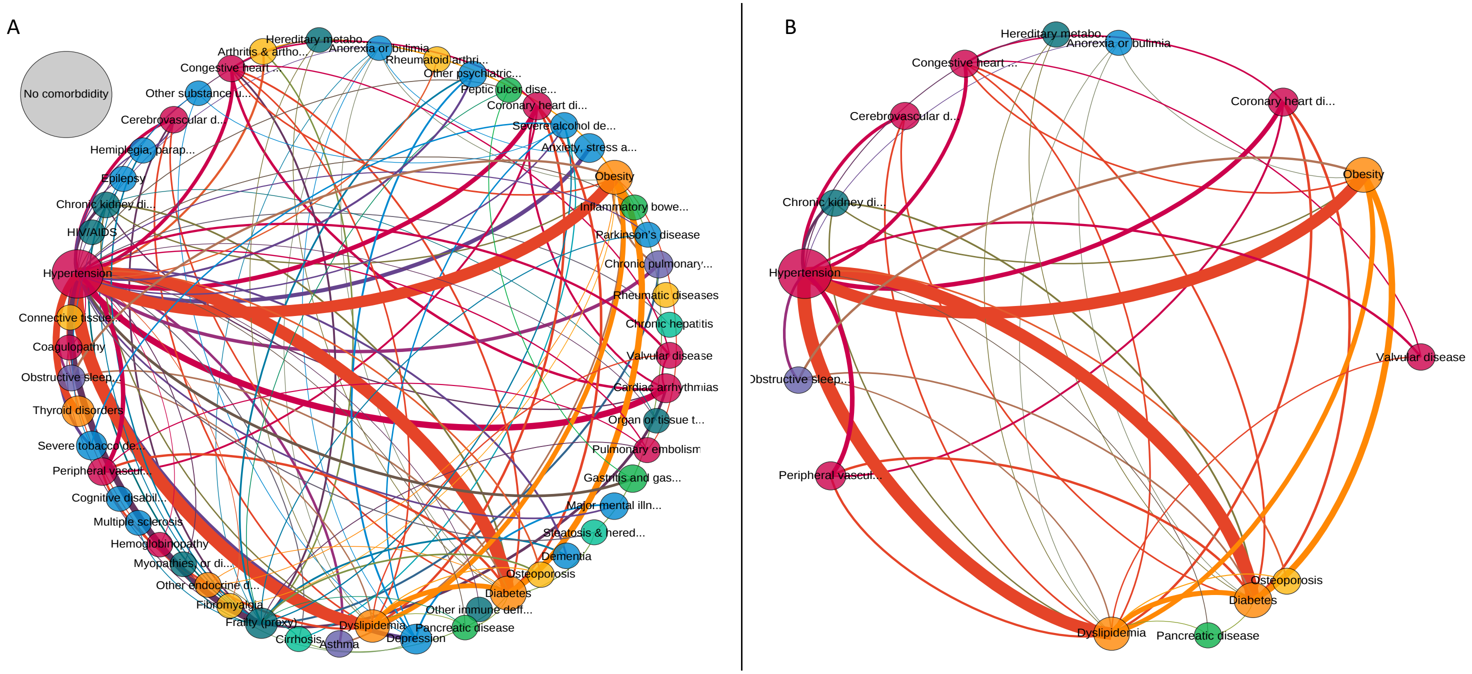

(A) General page screenshot. Nodes represent comorbid conditions (at disease level). Edges represent associations between comorbid conditions. An edge between two comorbidities is displayed only if at least 20% of the patients suffering from either of the two diseases also suffer from the other disease. Edge width is proportional to the number of patients suffering from both diseases. A node for patients without comorbid conditions was added for comparison. (B) Screenshot of the web page after clicking on "Dyslipidemia". Clicking on a node hides all nodes other than those connected to the selected disease, and all edges except those between the remaining nodes. Source data are provided as a Source Data file.
Abbreviations: HIV: human immunodeficiency virus, AIDS: acquired immunodeficiency syndrome.

Supplementary Figure 4: Association between medications (at ATC level 2) and comorbid conditions (at category level).

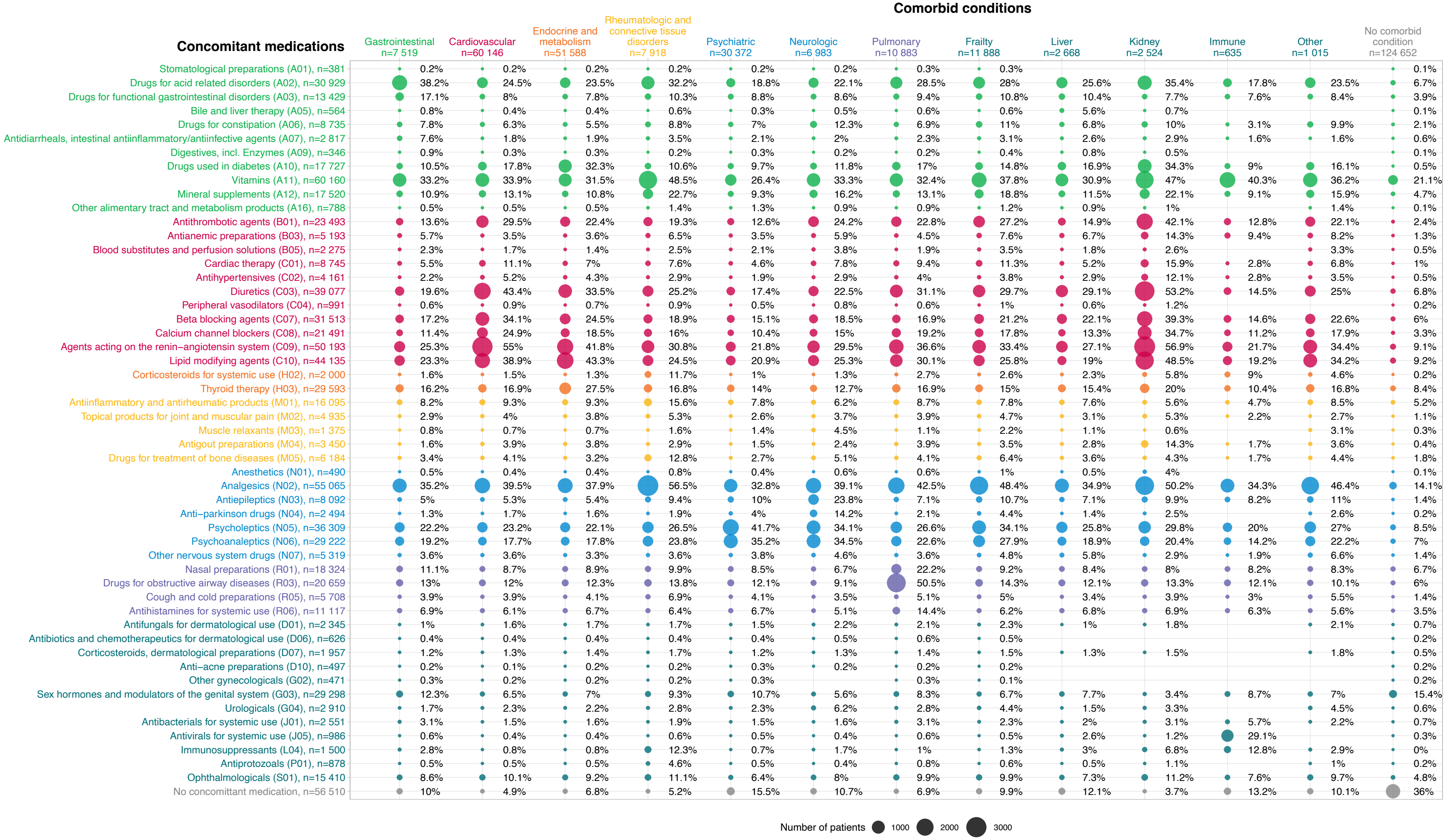

The size of the circle accounts for the percentage of patients suffering from the comorbid condition who used the medication at BC diagnosis; crude percentages of patients are displayed to the right of the circles. Circles are color-coded according to the ATC. Source data are provided as a Source Data file.

Supplementary Figure 5: Frequency and number of medications used at the time of BC diagnosis, for the total population, by deprivation index and by age at BC diagnosis.

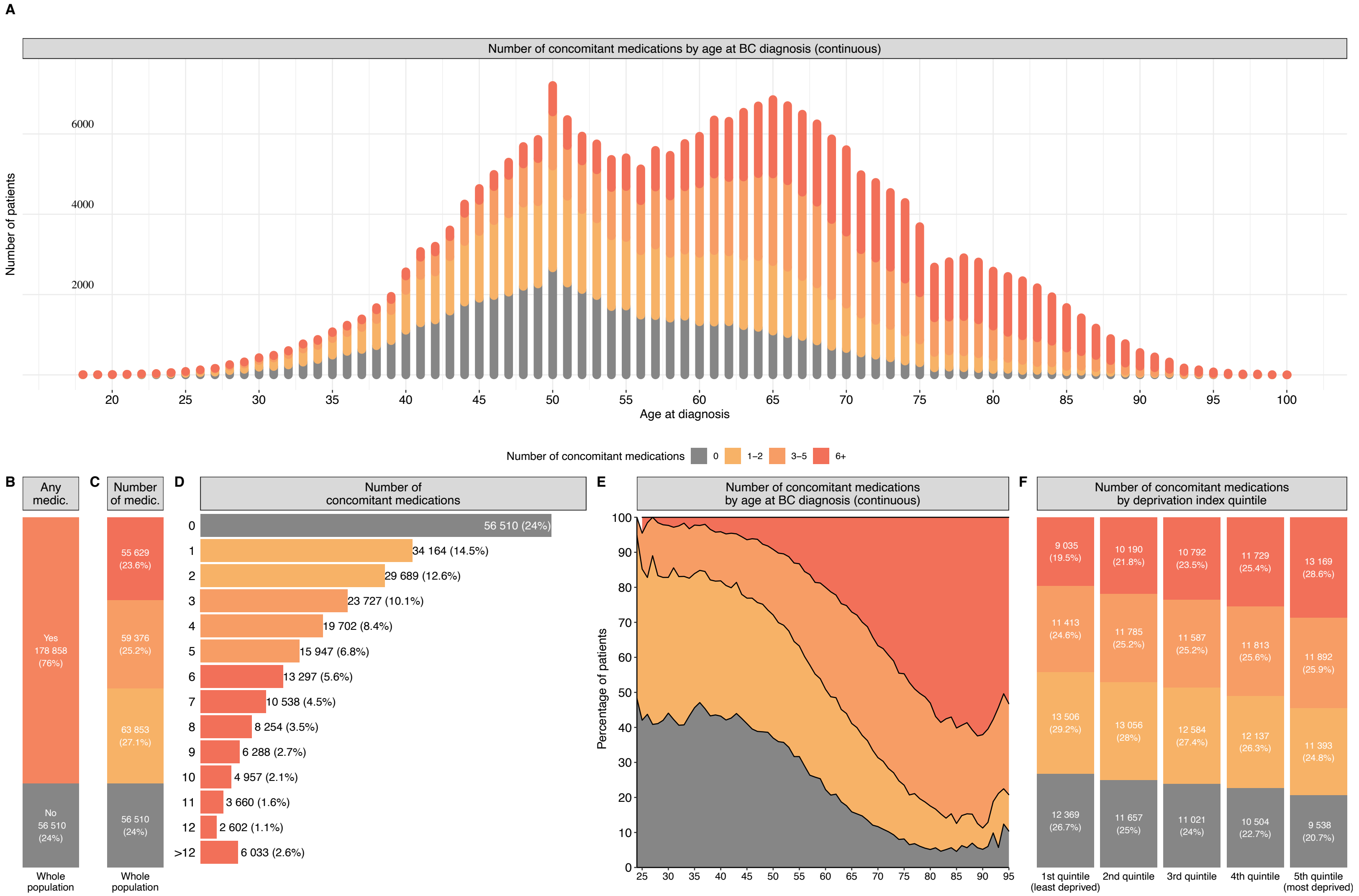

(A) Number of medications used concomitantly with BC diagnosis, by age at BC diagnosis; (B) Presence of concomitant medication use at BC diagnosis for the total population; (C) Number of concomitant medications, by class, for the total population; (D) Number of medications used at the time of BC diagnosis for the total population; (E) Number of medications used at the time of BC diagnosis, by age at BC diagnosis (as a percentage); (F) Number of medications used at the time of BC diagnosis, by deprivation index quintile. Patients from overseas départements with no data for deprivation index quintile (n=4,198) are not displayed in the figure. Source data are provided as a Source Data file.

Abbreviations: ATC: Anatomical Therapeutic Chemical.

Supplementary Figure 6: Association between medications (at ATC level 2).

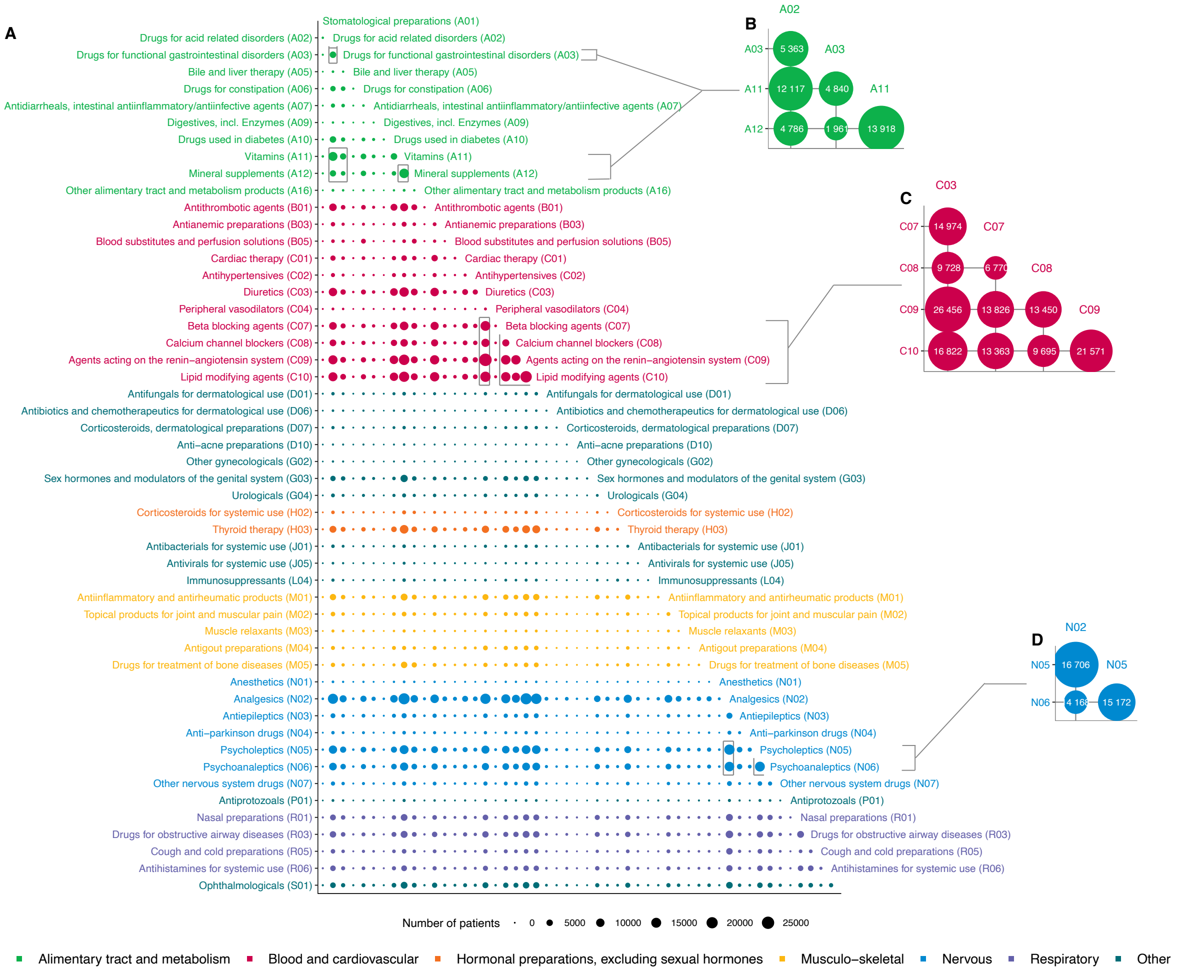

(A) Association between all ATC level 2 classes. The size of the circle is proportional to the number of patients using medications from both ATC level 2 classes. Comedications are color-coded according to their ATC level 1 category. (B) Associations between several selected frequently used ATC level 2 classes corresponding to ATC level 1 "A" (alimentary tract and metabolism), with crude numbers of patients. (C) Associations between several selected frequently used ATC level 2 classes corresponding to ATC level 1 "C" (cardiovascular system), with crude numbers of patients. (D) Associations between several selected frequently used ATC level 2 classes corresponding to ATC level 1 "N" (nervous system), with crude numbers of patients. Source data are provided as a Source Data file. Abbreviations: ATC: Anatomical Therapeutic Chemical.

Supplementary Figure 7: Screenshot of the web page for visualizing associations between medications at ATC level 5 within the ADRENALINE web application (https://adrenaline.curie.fr/static/network/index.html).

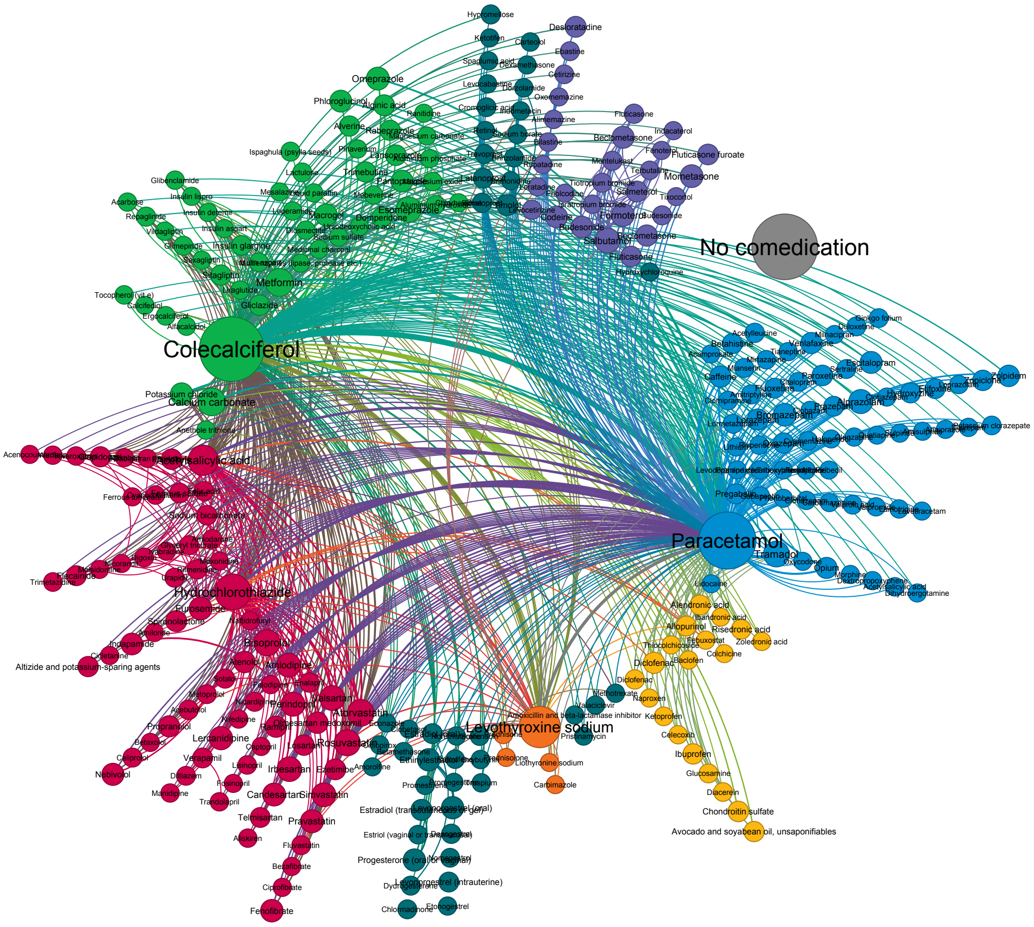

Nodes represent medications. Edges represent associations between medications. An edge between two medications (comedication) is displayed only if at least 20% of patients using one of the two medications also uses the other medication. Edge width is proportional to the number of patients using both medications. A node for patients without comedication has been added for comparison. Source data are provided as a Source Data file. Abbreviations: ATC: Anatomical Therapeutic Chemical.

Supplementary Figure 8: Association between the number of comorbid conditions and the number of medications.

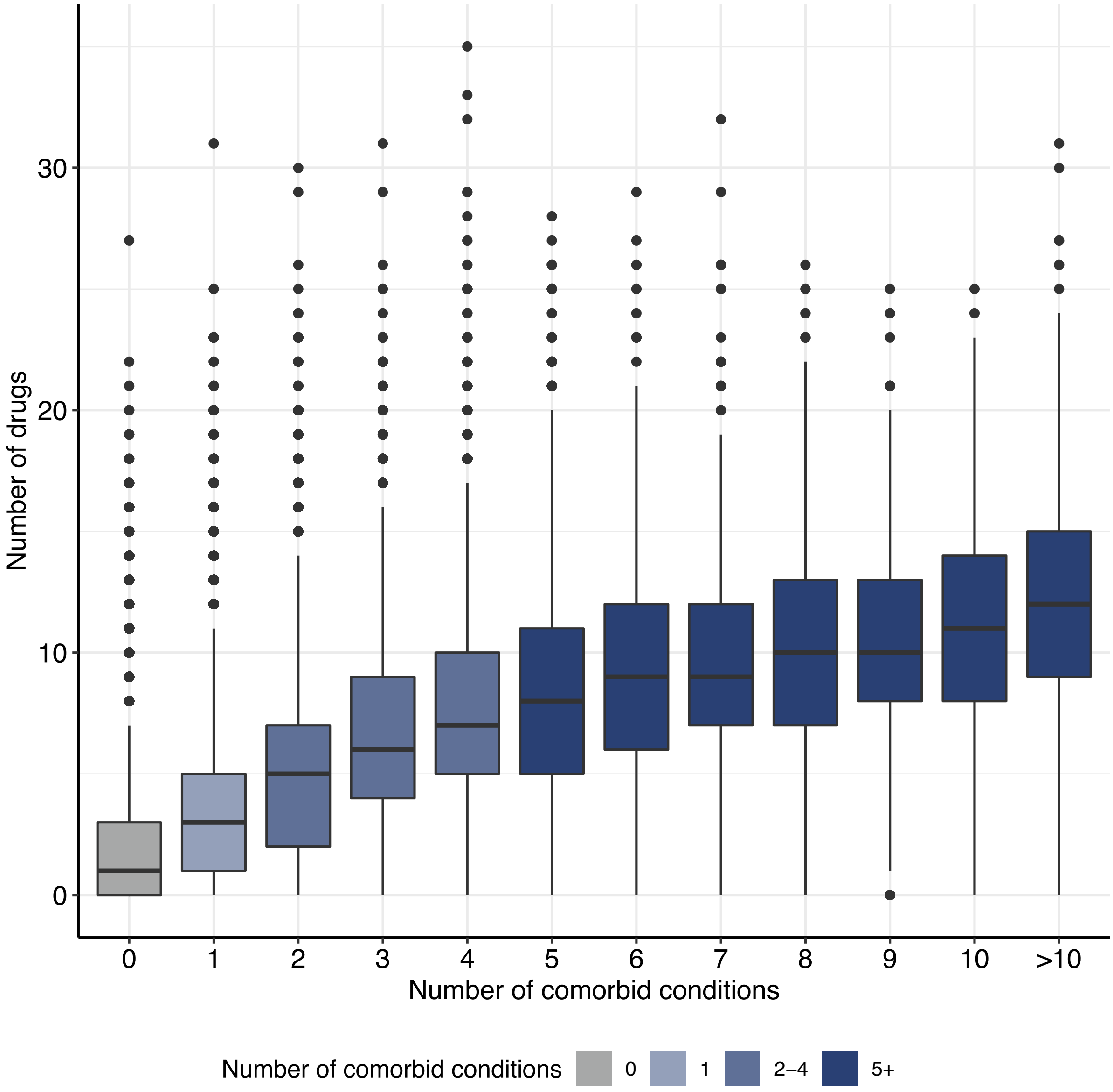

Boxplots are color-coded according to the number of comorbid conditions. The bars at the bottom and top of the boxplots represent the first and third quartiles, respectively. The bar in the middle corresponds to the median; and whiskers extend to 1.5 times the interquartile range Source data are provided as a Source Data file.

Supplementary Figure 9: Simplified flowchart of the French Early Breast Cancer Cohort.

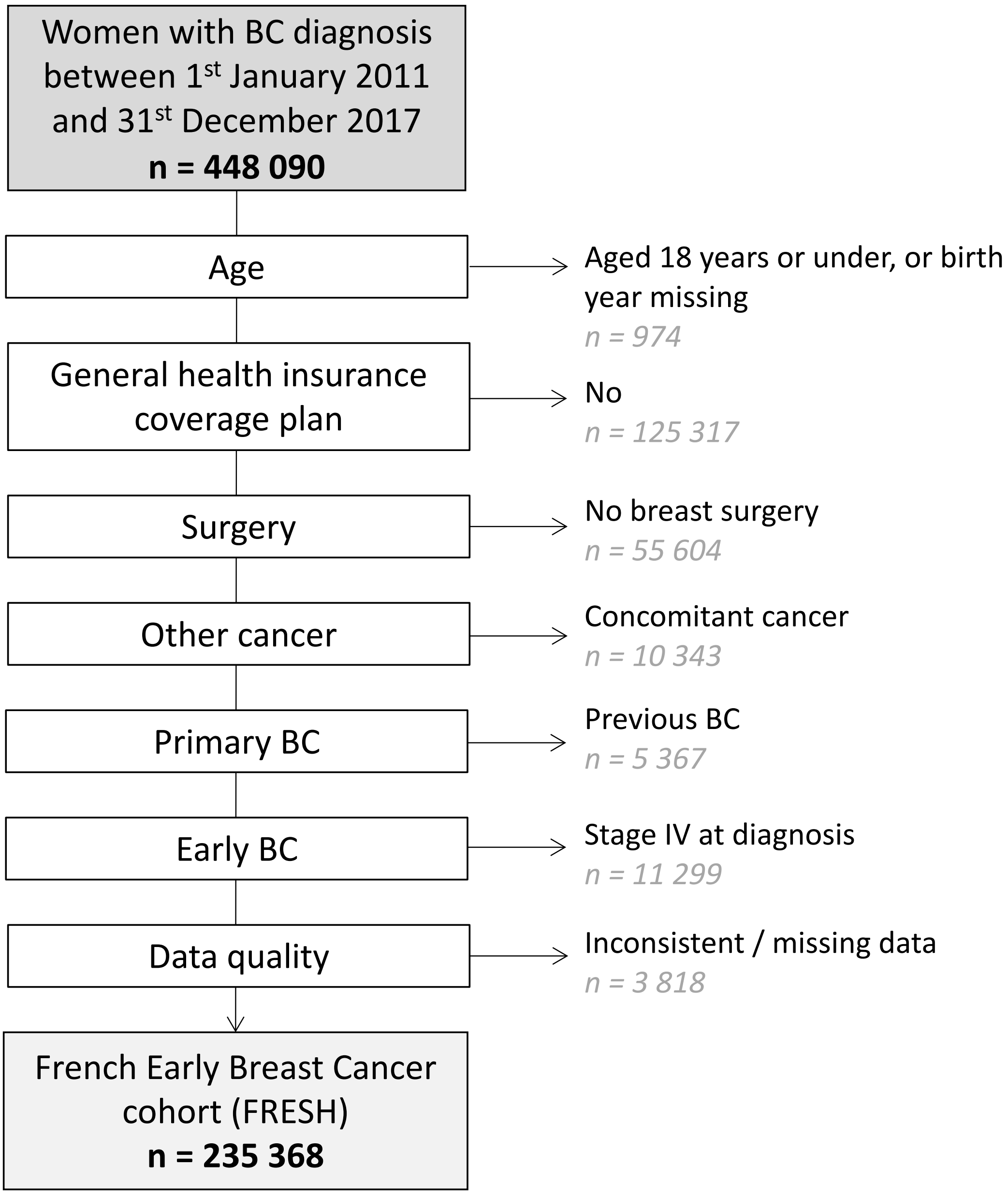

Abbreviations: BC: breast cancer.

Supplementary Figure 10: Modified ATC codes and ATC names for ATC class G03 (sex hormones and modulators of the genital system).

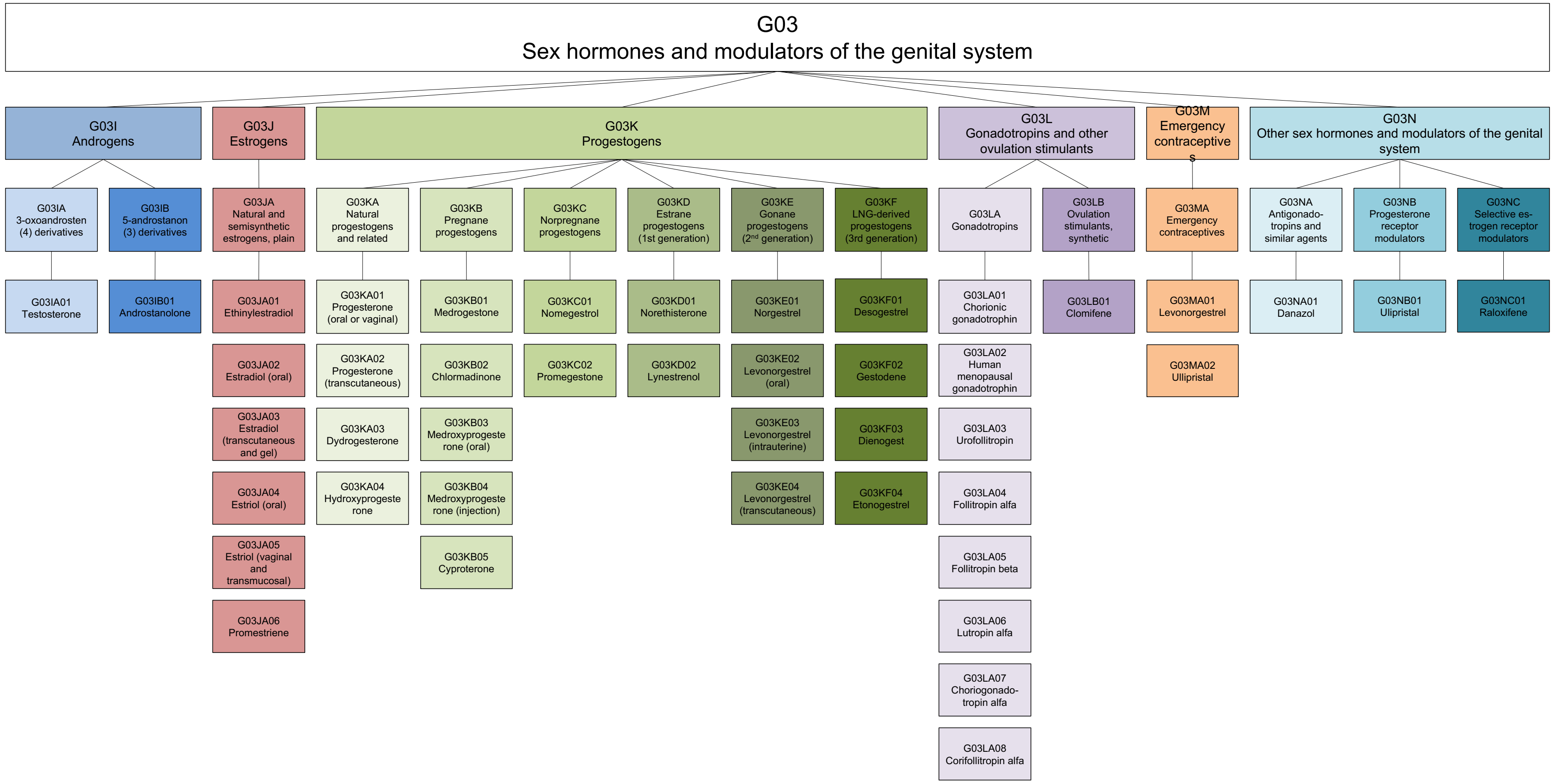

The first row corresponds to ATC level 2, the second row corresponds to ATC level 3, the third to ATC level 4, and the remaining rows (fourth to eleventh rows) to ATC level 5 (medications). The basic color assigned to each medication depends on ATC level 3, with the shade of the color thereafter depending on ATC level 4. *Abbreviations: ATC:* Anatomical Therapeutic Chemical.

Supplementary Figure 11: Directed acyclic graph (DAG).

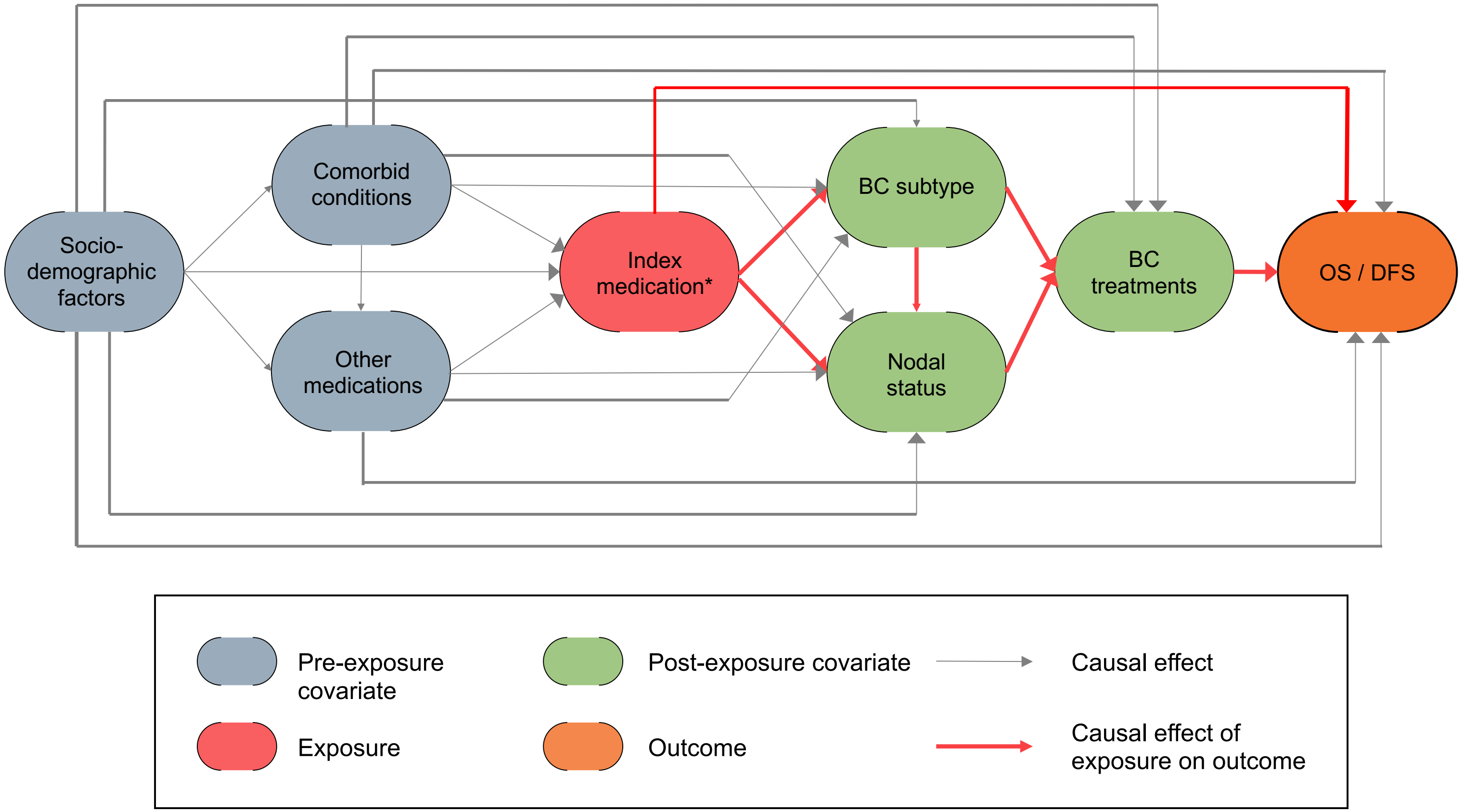

Nodes represent variables or group of variables included in the analyses. Directed edges between nodes represent causal effects. The goal of the study was to infer the average treatment effect (ATE) of each medication, or index medication (causal exposure, colored in red) on overall

survival and disease-free survival (causal outcomes, colored in orange). The average treatment effect (ATE) is the combination of all edges colored in red. The nodes of pre-exposure covariates are colored in blue. Pre-exposure covariates include: (1) sociodemographic factors (age, deprivation index, number of general practitioner visits and number of gynecologist visits in the year preceding breast cancer diagnosis); (2) comorbid conditions (52 diseases); and (3) comedication (concomitant use of a medication other than the index medication). Pre-exposure covariates were all considered to be potential confounders [21], as they were both predictors of medication intake and independent risk factors for the outcomes. The nodes of post-exposure covariates are colored in green. Post-exposure covariates included BC subtype, nodal status and BC treatment. Exposure to the index medication may influence BC biology at diagnosis. BC subtype and nodal status were therefore considered to be potential mediators of the effect of exposure on the outcomes. Given that all pre-exposure covariates were known, we assumed that exposure to the index medication did not influence the therapeutic choices of the clinicians. We, therefore, did not consider BC treatments to be mediators of the effect of exposure on the outcomes. * The index medication is the medication under investigation. *Abbreviations: BC: breast cancer; ATE: average treatment effect; ATC:* Anatomical Therapeutic Chemical; OS: overall survival; DFS: disease-free survival.

Supplementary Figure 12: Directed acyclic graph (DAG) for the mediation analyses.

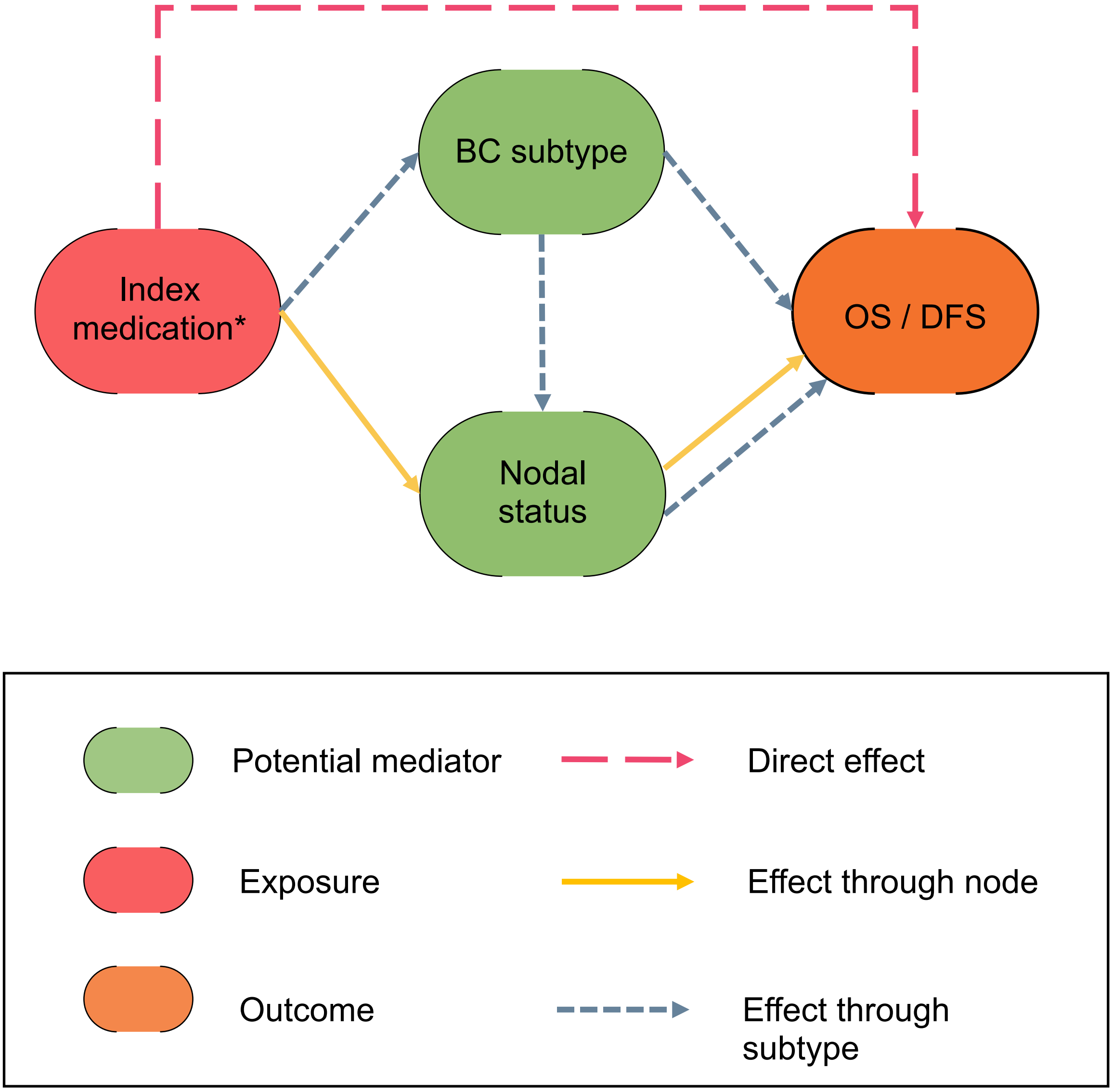

Nodes represent variables. Directed edges between nodes represent causal effects. The goal of the mediation analysis is to break down the average treatment effect (ATE) of the index medication (causal exposure; in red) on OS or DFS (causal outcomes, in orange) into three path-specific effects (PSEs) according to BC subtype and nodal status (potential mediators, in green). The arrows included in the three PSEs are color-coded as follows: (1) pink for the direct effect; (2) yellow for the effect through node (effect through nodal status and not through subtype); and (3) blue for the effect through subtype (effect through subtype and potentially through nodal status). We assumed that BC subtype was an independent risk factor for lymph node involvement, as reported elsewhere, so that there was a causal link between BC subtype and nodal status. * The index medication is the medication under investigation.

Only medications with a significant protective or deleterious ATE (hazard ratio) were selected for mediation analyses. *Abbreviations: BC: breast cancer; ATE: average treatment effect; ATC:* Anatomical Therapeutic Chemical*;* OS: overall survival; DFS: disease-free survival.

Supplementary References

1. World Health Organization. International Classification of Diseases (ICD). https://www.who.int/standards/classifications/classification-of-diseases.
2. Bannay, A. *et al.* The Best Use of the Charlson Comorbidity Index With Electronic Health Care Database to Predict Mortality. *Med Care* 54, 188–194 (2016).
3. Billioti de Gage, S. *et al.* Antidepressants and Hepatotoxicity: A Cohort Study among 5 Million Individuals Registered in the French National Health Insurance Database. *CNS Drugs* 32, 673–684 (2018).
4. Bouyer, B., Rudnichi, A., Dray-Spira, R., Zureik, M. & Coste, J. Thromboembolic risk after lumbar spine surgery: a cohort study on 325 000 French patients. *J Thromb Haemost* (2018) doi:10.1111/jth.14205.
5. Coste, J. *et al.* Risk of early neurodevelopmental disorders associated with in utero exposure to valproate and other antiepileptic drugs: a nationwide cohort study in France. *Sci Rep* 10, 17362 (2020).
6. Giral, P., Neumann, A., Weill, A. & Coste, J. Cardiovascular effect of discontinuing statins for primary prevention at the age of 75 years: a nationwide population-based cohort study in France. *Eur Heart J* 40, 3516–3525 (2019).
7. Jabagi, M. J. *et al.* Risk of secondary hematologic malignancies associated with breast cancer chemotherapy and G-CSF support: A nationwide population-based cohort. *International Journal of Cancer* n/a, (2020).
8. Lemaitre, M. *et al.* Association Between Use of Thiopurines or Tumor Necrosis Factor Antagonists Alone or in Combination and Risk of Lymphoma in Patients With Inflammatory Bowel Disease. *JAMA* 318, 1679–1686 (2017).

9. Maura, G. *et al.* Oral anticoagulants and risk of acute liver injury in patients with nonvalvular atrial fibrillation: a propensity-weighted nationwide cohort study. *Scientific Reports* 10, 11624 (2020).

10. Sbidian, E., Mezzarobba, M., Weill, A., Coste, J. & Rudant, J. Persistence of treatment with biologics for patients with psoriasis: a real-world analysis of 16 545 biologic-naïve patients from the French National Health Insurance database (SNIIRAM). *Br J Dermatol* 180, 86–93 (2019).

11. Semenzato, L. *et al.* Maladies chroniques, états de santé et risque d'hospitalisation et de décès hospitalier pour COVID-19 lors de la première vague de l'épidémie en France: Étude de cohorte de 66 millions de personnes. 41 (2021).

12. Dumas, E. *et al.* The French Early Breast Cancer Cohort (FRESH): a resource for breast cancer research and evaluations of oncology practices based on the French National Healthcare System Database (SNDS). 2022.03.14.22272286 Preprint at https://doi.org/10.1101/2022.03.14.22272286 (2022).

13. Rey, G., Jougla, E., Fouillet, A. & Hémon, D. Ecological association between a deprivation index and mortality in France over the period 1997 - 2001: variations with spatial scale, degree of urbanicity, age, gender and cause of death. *BMC Public Health* 9, 33 (2009).

14. Austin, P. C. An Introduction to Propensity Score Methods for Reducing the Effects of Confounding in Observational Studies. *Multivariate Behav Res* 46, 399–424 (2011).

15. Austin, P. C. & Stuart, E. A. Moving towards best practice when using inverse probability of treatment weighting (IPTW) using the propensity score to estimate causal treatment effects in observational studies. *Stat Med* 34, 3661–3679 (2015).

16. Cole, S. R. & Hernán, M. A. Constructing Inverse Probability Weights for Marginal Structural Models. *Am J Epidemiol* 168, 656–664 (2008).

17. VanderWeele, T. J., Vansteelandt, S. & Robins, J. M. Effect decomposition in the presence of an exposure-induced mediator-outcome confounder. *Epidemiology* 25, 300–306 (2014).

18. Vanderweele, T. J. *Explanation in Causal Inference: Methods for Mediation and Interaction*. xvi, 706 (Oxford University Press, New York, NY, US, 2015).

19. Chatton, A. *et al.* G-computation, propensity score-based methods, and targeted maximum likelihood estimator for causal inference with different covariates sets: a comparative simulation study. *Sci Rep* 10, 9219 (2020).

20. Le Borgne, F., Giraudeau, B., Querard, A. H., Giral, M. & Foucher, Y. Comparisons of the performance of different statistical tests for time-to-event analysis with confounding factors: practical illustrations in kidney transplantation. *Stat Med* 35, 1103–1116 (2016).

21. Brookhart, M. A. *et al.* Variable selection for propensity score models. *Am J Epidemiol* 163, 1149–1156 (2006).
