# Supplementary material for: Concomitant medication, comorbidity and survival in patients with breast cancer
Source: Nat Commun. 2024 Apr 5;15:2966. doi: 10.1038/s41467-024-47002-3 (PMC10997660; doi:10.1038/s41467-024-47002-3)
Supplement: Supplementary file 3 — Description of additional supplementary files [file 41467_2024_47002_MOESM3_ESM.pdf]

Description of Additional Supplementary Files

File: Supplementary Data 1
Description: Number of medication users by ATC (Anatomical Therapeutic Chemical) level (1, 2, 3, 4 and 5).

File: Supplementary Data 2
Description: Estimated average treatment effect (ATE, Cox hazard ratio), along with its 95% confidence interval for overall survival (OS), and p-value for the 113 medications passing the adjustment quality test. Abbreviations: ATC: Anatomical Therapeutic Chemical; CI: confidence interval; Systemic hormonal preparations*: systemic hormonal preparations, excluding sex hormones and insulins.

File: Supplementary Data 3
Description: Estimated average treatment effect (ATE, Cox hazard ratio), along with its 95% confidence interval for disease-free survival (DFS), and p-value for the 113 medications passing the adjustment quality test. Abbreviations: ATC: Anatomical Therapeutic Chemical; CI: confidence interval; Systemic hormonal preparations*: systemic hormonal preparations, excluding sex hormones and insulins.

File: Supplementary Data 4
Description: List of ICD10, CCAM, LPP and NGAP codes used to identify comorbid conditions. Abbreviations: ICD10: International Classification of Diseases – 10th revision, CCAM: “Classification Communes des Actes Médicaux”, LPP: “Liste des produits et prestations”, NGAP: “Nomemenclature Générale des Actes Professionnels”, LTI: long-term illness, HIV: human immunodeficiency virus, AIDS: acquired immunodeficiency syndrome; ATC: Anatomical Therapeutic Chemical.

File: Supplementary Data 5
Description: List of CCAM, ICD-10 and ATC codes used to identify breast cancer relapses. Abbreviations: ICD 10: international statistical classification and related health problems – 10th revision; CCAM: “Classification Communes des Actes Médicaux”; ATC = anatomical therapeutic and chemical classification.
