# Supplementary material for: Concomitant medication, comorbidity and survival in patients with breast cancer
Source: Nat Commun. 2024 Apr 5;15:2966. doi: 10.1038/s41467-024-47002-3 (PMC10997660; doi:10.1038/s41467-024-47002-3)
Supplement: Supplementary file 9 — Reporting Summary [file 41467_2024_47002_MOESM9_ESM.pdf]

nature portfolio

Corresponding author(s): Prof. Fabien REYAL

Last updated by author(s): 18/03/2024

Reporting Summary

Nature Portfolio wishes to improve the reproducibility of the work that we publish. This form provides structure for consistency and transparency in reporting. For further information on Nature Portfolio policies, see our Editorial Policies and the Editorial Policy Checklist.

Statistics

For all statistical analyses, confirm that the following items are present in the figure legend, table legend, main text, or Methods section.

n/a	Confirmed	
☐	☒	The exact sample size (*n*) for each experimental group/condition, given as a discrete number and unit of measurement
☐	☒	A statement on whether measurements were taken from distinct samples or whether the same sample was measured repeatedly
☐	☒	The statistical test(s) used AND whether they are one- or two-sided<br>*Only common tests should be described solely by name; describe more complex techniques in the Methods section.*
☐	☒	A description of all covariates tested
☐	☒	A description of any assumptions or corrections, such as tests of normality and adjustment for multiple comparisons
☐	☒	A full description of the statistical parameters including central tendency (e.g. means) or other basic estimates (e.g. regression coefficient) AND variation (e.g. standard deviation) or associated estimates of uncertainty (e.g. confidence intervals)
☐	☒	For null hypothesis testing, the test statistic (e.g. *F*, *t*, *r*) with confidence intervals, effect sizes, degrees of freedom and *P* value noted<br>*Give P values as exact values whenever suitable.*
☐	☒	For Bayesian analysis, information on the choice of priors and Markov chain Monte Carlo settings
☐	☒	For hierarchical and complex designs, identification of the appropriate level for tests and full reporting of outcomes
☐	☒	Estimates of effect sizes (e.g. Cohen's *d*, Pearson's *r*), indicating how they were calculated

Our web collection on statistics for biologists contains articles on many of the points above.

Software and code

Policy information about availability of computer code

Data collection	No software was used.
Data analysis	Analyses were performed with R software, version 3.6.3. Propensity scores were computed with the glm function from the R base package stats. Standardized mean differences (SMDs) were computed with the bal.tab function from the R package cobalt (version 4.1.0). Cox proportional hazards models were fitted with the coxph function from the R package survival (version 3.2-3). Weighted Kaplan-Meier estimators were obtained with the R package RISCA (version 0.8.2). Survival curves were drawn with the R package survminer (version 0.4.7). The web application was built with Flask version 2.0.2, using Python version 3.9.1.Codes will be available at https://github.com/rt2lab/adrenaline prior to publication.

For manuscripts utilizing custom algorithms or software that are central to the research but not yet described in published literature, software must be made available to editors and reviewers. We strongly encourage code deposition in a community repository (e.g. GitHub). See the Nature Portfolio guidelines for submitting code & software for further information.

Data

Policy information about availability of data

All manuscripts must include a data availability statement. This statement should provide the following information, where applicable:

- Accession codes, unique identifiers, or web links for publicly available datasets
- A description of any restrictions on data availability
- For clinical datasets or third party data, please ensure that the statement adheres to our policy

Data available: no.
Justification: According to data protection and French regulation, the authors cannot publicly release the data from the SNDS. However, any person or structure, public or private, for-profit or nonprofit, is able to access SNDS data upon authorization from the French Data Protection Office (CNIL, Commission Nationale de l'Informatique et des Libertés) to carry out a study, a research, or an evaluation of public interest.

Research involving human participants, their data, or biological material

Policy information about studies with human participants or human data. See also policy information about sex, gender (identity/presentation), and sexual orientation and race, ethnicity and racism.

Reporting on sex and gender	The study only considered female patients, based on their civil status, as available in the SNDS data.
Reporting on race, ethnicity, or other socially relevant groupings	Race and ethnicity were not collected or analyzed. Socioeconomic status was proxied only by the deprivation index of the area of residence. Confounders were adjusted using inverse probability weighted marginal structural models.
Population characteristics	We included all women diagnosed with early-stage BC between January 1, 2011 and December 31, 2017 in France, treated by surgery, and registered in the general health insurance coverage plan. Patients with suspected distant metastases or concomitant cancer of another localization at diagnosis were excluded.
Recruitment	In accordance with French regulations applicable to the SNDS data, no individualized consent was required because the data used in the study was de-identified and re-used for research purposes. Nevertheless, individuals have the right to refuse the use of their personal data contained in the SNDS for research purposes, and can exercise this right by opting-out. In such cases, their data was excluded from the analysis. The study may be subject to selection biases as it was restricted to patients who did not refuse the reuse of their data.
Ethics oversight	The study was authorized by the French data protection agency (Commission nationale de l'informatique et des libertés—CNIL, under registration number 920017).

Note that full information on the approval of the study protocol must also be provided in the manuscript.

Field-specific reporting

Please select the one below that is the best fit for your research. If you are not sure, read the appropriate sections before making your selection.

☒ Life sciences ☐ Behavioural & social sciences ☐ Ecological, evolutionary & environmental sciences

For a reference copy of the document with all sections, see nature.com/documents/nr-reporting-summary-flat.pdf

Life sciences study design

All studies must disclose on these points even when the disclosure is negative.

Sample size	We analyzed the data of all individuals available in the SNDS data who respected the inclusion/exclusion criteria.
Data exclusions	No data was excluded from analyses.
Replication	No replication was available.
Randomization	Randomization was not applicable in this retrospective nationwide cohort study.
Blinding	Participants were not allocated to experimental groups during recruitment, data preprocessing and statistical analyses. Blinding was therefore not applicable.

Reporting for specific materials, systems and methods

We require information from authors about some types of materials, experimental systems and methods used in many studies. Here, indicate whether each material, system or method listed is relevant to your study. If you are not sure if a list item applies to your research, read the appropriate section before selecting a response.

Materials & experimental systems

n/a	Involved in the study
☒	☐ Antibodies
☒	☐ Eukaryotic cell lines
☒	☐ Palaeontology and archaeology
☒	☐ Animals and other organisms
☒	☐ Clinical data
☒	☐ Dual use research of concern
☒	☐ Plants

Methods

n/a	Involved in the study
☒	☐ ChIP-seq
☒	☐ Flow cytometry
☒	☐ MRI-based neuroimaging

Plants

Seed stocks	Not applicable
Novel plant genotypes	Not applicable
Authentication	Not applicable
